# To the Editors of the Medical and Physical Journal

**Published:** 1805-11-01

**Authors:** Thomas Pope

**Affiliations:** Cleobury, Salop


### *To the Editors of the Medical and Physical Journal.*

GENTLEMEN,

ON Saturday evening last I was sent for to a man dangerously ill, as the messenger expressed himself; and when I reached my patient, about eight o'clock, I found the expression but too well founded, for he was labouring under an incarcerated hernia, (oscheocele.)

The descent took place (not for the first time) about three o'clock the same day, and the pain soon became excessive, attended with vomiting, transitory delirium, cold sweat, and tormina in the bowels.

Judging the case to be of the most desperate nature, and in a plethoric and robust frame, after ineffectual attempts to reduce it by the taxis, I bled him to the amount of xxxv oz. and from a large orifice.

On repeating my efforts with a little force, the bowel receded from my hand, and returned, reluctant and grumbling, within its proper confines.

On giving him an aperient, and applying a truss, the subsequent day being the Sabbath, he spent part of it, as all in this Christian country ought, at church.

Having heard of the neglect of venæsection in a late similar case, which had a fatal termination, induced me to transmit you this, at the same time submitting to you the propriety of its insertion in your interesting Miscellany. I am, &c.

*Cleobury, Salop, Sept.* 10, 1805. THOMAS POPE.

---

